# A Rare Case of Giant Leiomyoma in the Space of Retzius

**DOI:** 10.1155/crog/5529983

**Published:** 2026-01-07

**Authors:** Emma Barr, Alireza Abidi

**Affiliations:** ^1^ Department of Obstetrics and Gynecology, Adventist Health White Memorial, Los Angeles, California, USA, adventisthealth.org; ^2^ Los Angeles Cancer Network, Riverside, California, USA

## Abstract

Leiomyoma are benign tumors of smooth muscle origin. Uterine leiomyoma are extremely common; leiomyoma in extraperitoneal spaces, however, are not. There have been several documented cases of leiomyoma in retroperitoneal spaces, though the majority of them have been in the posterior retroperitoneum. Here, we report a patient presenting with a large, firm suprapubic mass, initially thought to be a fundal uterine fibroid. On surgical excision, it was found to be the extremely rare finding of a benign leiomyoma in the anterior retroperitoneum or space of Retzius.

## 1. Background

Leiomyomas are benign, monoclonal tumors that arise from smooth muscle tissue [1]. Uterine leiomyomas (or uterine fibroids) are the most common uterine tumors, with approximately 70% of white women and 80% of Black women having at least one leiomyoma by the age of 50 [1, 2]. On pathological inspection, 90% of leiomyoma fall into the most common type of leiomyoma (“conventional” or “usual” leiomyoma) and have several essential features, including monotonous spindle cells with indistinct borders arranged in intersecting fascicles and rare mitoses [2]. Leiomyomas most commonly occur in the uterine corpus; however, they can occur in other gynecologic organs, including the ovary, broad ligament, cervix, vagina, and vulva [3]. Rarely, leiomyoma can be found in extraperitoneal spaces, such as the retroperitoneum [4]. The vast majority of retroperitoneal leiomyoma are found in the posterior retroperitoneum; only a few have been documented to occur in the anterior retroperitoneum or space of Retzius. Here, we report a 23‐year‐old presenting with a lower abdominal mass and pelvic pressure, found to have a large, extraperitoneal leiomyoma in the space of Retzius.

## 2. Case Presentation

**Figure 1:**
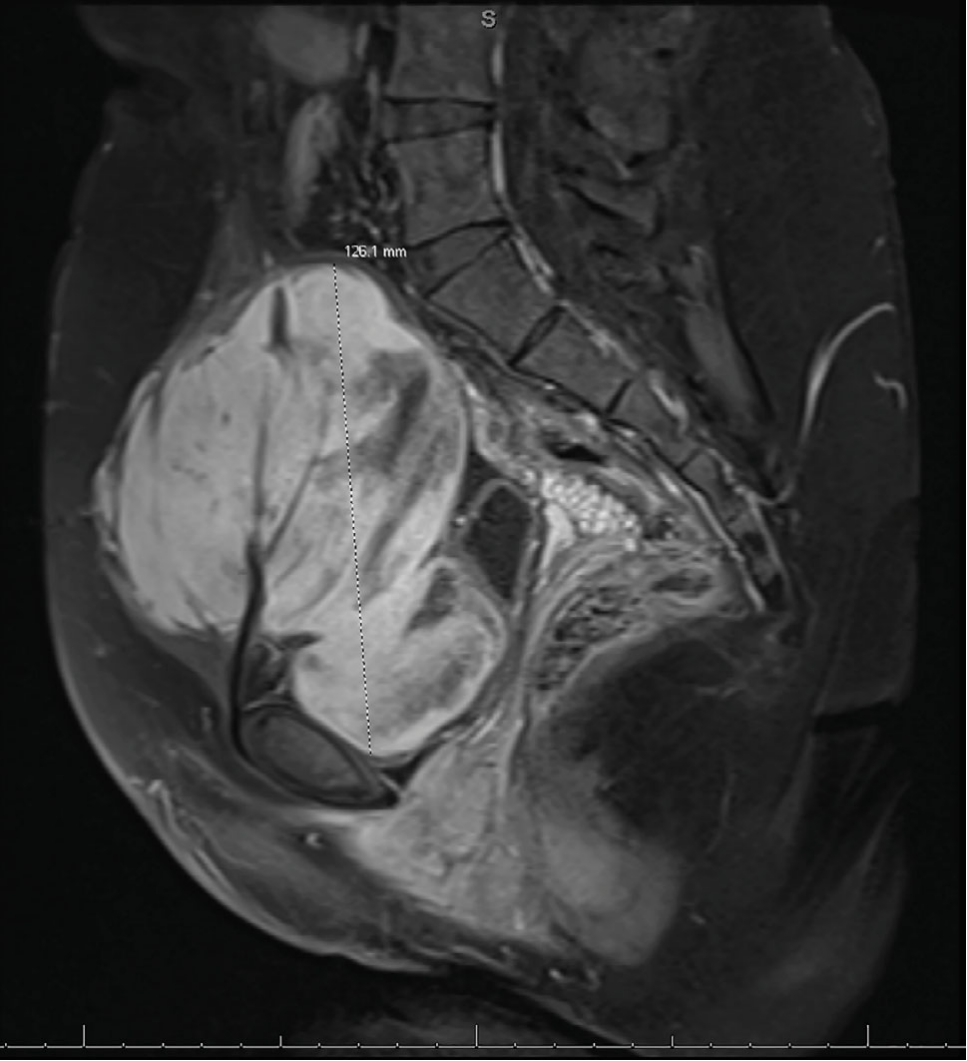
MRI pelvis demonstrating the prominent vascularity supplying the mass, arising from the pubic symphysis region, possibly the inferior epigastric artery.

**Figure 2:**
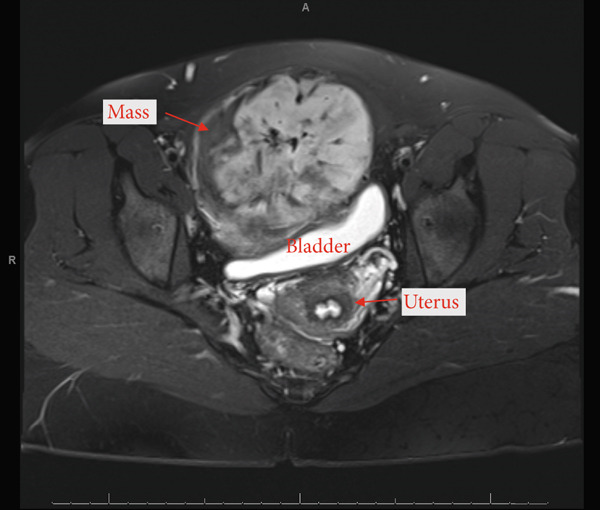
MRI pelvis demonstrating the mass to be distinct from the bladder and uterus.

A 23‐year‐old gravida 1, para 1 female was referred to gynecologic oncology from her primary care physician for a complex pelvic mass enlarging over the past 4 years causing pressure on her bladder. The patient had no past medical or surgical history. Obstetric history was significant for one normal spontaneous vaginal delivery. On abdominal exam, there was a large, very firm, and immobile suprapubic abdominal mass, at least 15 cm in size. Pelvic exam was unremarkable with an approximately 12‐week sized, mobile uterus and no appreciable adnexal masses. The mass was initially presumed to be a large fundal fibroid based off transvaginal ultrasound; however, magnetic resonance imaging (MRI) revealed a 12.6 × 10.2 × 9.1 cm, T1 hypointense and T2 hyperintense mass located in the anterior aspect of the pelvis. There was prominent vascularity supplying the mass, arising from the pubic symphysis region, possibly the inferior epigastric artery (Figure 1). The lesion was noted to cause mass effect on the bladder, uterus, and cervix, displacing them posteriorly. There did not appear to be a distinct connection between the mass and the uterus (Figure 2). The uterus and ovaries were otherwise normal appearing.

The patient underwent exploratory laparotomy via vertical midline incision and radical excision of the pelvic tumor. Intraoperative findings revealed a 14 cm solid mass in the space of Retzius, adhered to the rectus fascia and pubic symphysis. Normal uterus, ovaries, and bilateral tubes were noted. The mass was resected, removed, and sent to pathology, which was consistent with leiomyoma. The patient did well postoperatively and was discharged home on postoperative day 1. She has since recovered uneventfully. On the final pathological report, the mass weighed 630 grams and measured 14 × 14 × 9.2 cm (Figure 3). The final histopathological diagnosis was spindle cell lesion, favor leiomyoma, negative for malignancy.

Figure 3Gross specimen of the leiomyoma in the space of Retzius. (a) Right lateral aspect of the mass. (b) Posterior aspect of the mass.

**Figure:**
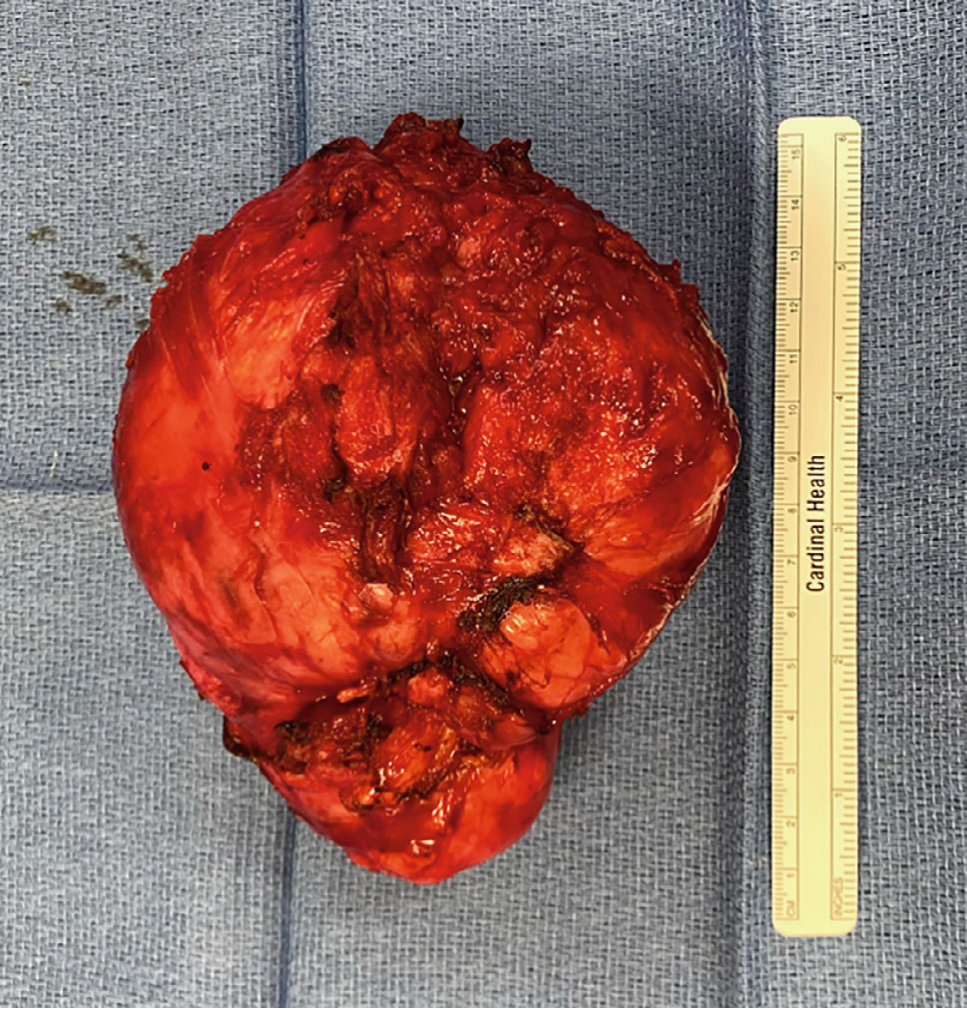
(a)

**Figure:**
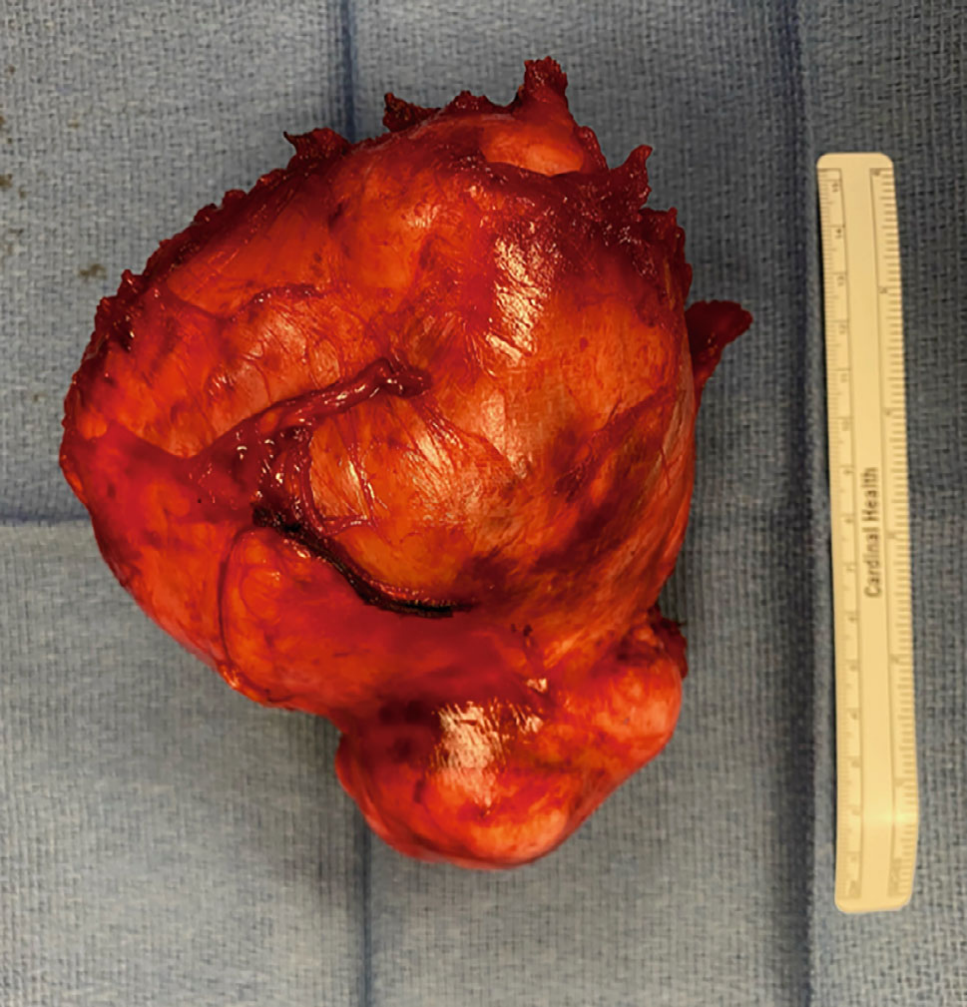
(b)

## 3. Discussion

As previously stated, uterine leiomyoma are an extremely common condition; leiomyoma in the space of Retzius, however, are not. Here, we present the twelfth documented case of a leiomyoma in the space of Retzius [5]. Most of the other documented cases ranged in size from 2 to 10 cm, while the mass in our patient was 14 cm [6].

Other authors have postulated that these leiomyoma in the space of Retzius arise from either iatrogenic, embryologic, or anatomic etiologies. Iatrogenic seeding of the space of Retzius could theoretically take place during hysterectomy or myomectomy, performed via laparotomy or minimally invasively surgery, especially if morcellation was performed. Unfortunately, most of the prior reports of leiomyoma in the space of Retzius did not include the patient’s surgical history—it is therefore hard to state if there has been a correlation in history of surgery and these tumors [4, 6–9]. Our patient had no history of prior surgeries so iatrogenic etiology is impossible in this case.

Alternatively, Stutterecker et al., who reported on the first two cases of leiomyoma in the space of Retzius, suggested that leiomyoma in the pubovesical space could arise from embryonal (Mullerian or Wolffian) duct remnants or from the local vessel musculature [10]. Interestingly, the mass in our patient was noted to have a strikingly prominent vessel on MRI arising from the pubic symphysis region, possibly the inferior epigastric artery. This finding could support the hypothesis that these benign smooth muscle cell tumors actually arise from the smooth muscle cells in the retropubic space, not the smooth muscle cells of the uterus.

Overall, this case exemplifies several key learning points to keep in mind when assessing and managing large pelvic masses. First, multiple imaging modalities helped determine the origin of the mass in this case, which helped guide the selection of the appropriate surgeon and approach. Initially, the mass was thought to be a fundal fibroid on ultrasound; however, with MRI imaging, it was discovered that the mass was less likely to be attached to any of the gynecologic organs. This informed the decision to have gynecologic oncology perform the surgery, given the potential need for additional staging procedures and/or the possibility of extremely distorted anatomy requiring complex surgical techniques. Furthermore, as there is a growing number of case reports on leiomyoma in the space of Retzius, it is important to report on the patient’s surgical history, as well as any unique imaging findings (such as the prominent feeding vessel in our case), to help narrow down and ultimately discover the true etiology of these leiomyoma that are distinct and unconnected from the uterus.

## Consent

Written informed consent was obtained from the patient for publication of this case report and accompanying images. A copy of the written consent is available for review by the Editor‐in‐Chief of this journal on request.

## Conflicts of Interest

The authors declare no conflicts of interest.

## Funding

No funding was received for this manuscript.

## Data Availability

Data sharing is not applicable to this article as no new data were created or analyzed in this study.
